# Financial Toxicity, Hope, and Satisfaction With Life in Patients Receiving Ambulatory Cancer Care

**DOI:** 10.1001/jamanetworkopen.2025.57328

**Published:** 2026-02-05

**Authors:** Grace L. Smith, David B. Feldman, Hilary Ma, Christina Checka, Michael E. Roth, James C. Tucker, Cynthia Anderson, Marin Xavier, Jodi Kagihara, Ethan B. Ludmir, Chi-Fang Wu, Edna Paredes, Kathrin Milbury, Benjamin W. Corn

**Affiliations:** 1 Department of GI Radiation Oncology, MD Anderson Cancer Center, Houston, Texas; 2 Department of Health Services Research, MD Anderson Cancer Center, Houston, Texas; 3 Department of Counseling Psychology, Santa Clara University, Santa Clara, California; 4 Faculty of Psychology, Chulalongkorn University, Bangkok, Thailand; 5 Department of General Medical Oncology, MD Anderson Cancer Center, Houston, Texas; 6 Department of Breast Surgical Oncology, MD Anderson Cancer Center, Houston, Texas; 7 Department of Pediatrics, MD Anderson Cancer Center, Houston, Texas; 8 Lewis and Faye Cancer Center at DCH Regional Medical Center, Tuscaloosa, Alabama; 9 Baptist MD Anderson Cancer Center, Jacksonville, Florida; 10 Scripps Cancer Center, San Diego, California; 11 The Queen’s Medical Center, Honolulu, Hawaii; 12 Department of Biostatistics, MD Anderson Cancer Center, Houston, Texas; 13 Division of Radiation Oncology, MD Anderson Cancer Center, Houston, Texas; 14 Health Services Research, MD Anderson Cancer Center, Houston, Texas; 15 Behavioral Science, MD Anderson Cancer Center, Houston, Texas; 16 Department of Oncology, Hebrew University Faculty of Medicine, Jerusalem, Israel

## Abstract

**Importance:**

Financial toxicity significantly affects individuals with cancer, impacting not only treatment adherence and outcomes but also psychological dimensions such as satisfaction with life (SWL). Identifying mediators of psychological outcomes of financial toxicity, including hopefulness and social support, is critical for informing interventions to mitigate financial toxicity burdens.

**Objective:**

To examine the association of financial toxicity with hopefulness, social support, and SWL in patients with cancer, and to test the roles of hopefulness and social support in the association between financial toxicity and SWL.

**Design, Setting, and Participants:**

This cross-sectional analysis assessed baseline patient-reported financial toxicity from participants of the prospective, multisite Economic Strain and Resilience in Cancer-II (ENRICh-II) study who enrolled from August 2020 to December 2022. Included patients were adults receiving ambulatory cancer care for various malignant neoplasm types diagnosed in the year prior from academic, community-based, and federally qualified health centers across 6 oncology clinics. Data were primarily analyzed between January and June 2024.

**Exposure:**

Financial toxicity was assessed using the ENRICh instrument, which measures global financial toxicity burden and financial toxicity–related material, coping, and psychological distress burden subdomain scores.

**Main Outcomes and Measures:**

The Satisfaction with Life Scale measured life satisfaction. Hypothesized mediators were measured using the State Hope Scale and Medical Outcomes Study Social Support Survey. A bootstrapping approach to multiple mediation was applied, controlling for demographic and clinical covariates.

**Results:**

A total of 519 participants were surveyed (mean [SD] age, 52.0 [15.2] years; 349 female [67.2%]; 49 Black [9.4%], 137 Hispanic [26.4%], 278 White [53.6%]). The most common cancers included breast (202 [38.9%]); colon, rectum, and anal (111 [21.4%]); biliary, liver, pancreatic, and stomach (65 [12.5%]); and leukemia, lymphoma, and other hematologic malignant neoplasms (53 [10.2%]). Higher financial toxicity was significantly associated with lower SWL (*r* = −0.34, *P* < .001). Hopefulness and social support each partially mediated this association (social support, −0.03 [95% CI, −0.06 to −0.01]; hope, −0.08, [95% CI, −0.12 to −0.03]). The combined multiple mediation path was significant (−0.02 [95% CI, −0.04 to −0.01]). These associations also existed for the material, coping, and psychological distress financial toxicity domains.

**Conclusions and Relevance:**

In this multisite, cross-sectional study, greater financial toxicity burden was associated with lower life satisfaction; individuals’ hopefulness and perceived social support levels—representing dimensions of psychosocial resilience—statistically mediated this association. These results support an approach that would embed strategies to strengthen psychosocial resilience factors, in combination with current financial assistance, to help mitigate the downstream impacts of cancer-related financial toxicity.

## Key Points

**Question:**

Is financial toxicity associated with lower life satisfaction in patients with cancer, and do psychological resources (hopefulness and social support) explain this association?

**Findings:**

In 519 patients with cancer, financial toxicity burden was associated with lower life satisfaction (*r* = −0.34, *P* < .001). This association was partially explained by lower hopefulness (−0.08 [95% CI, −0.12 to −0.03]) and less social support (−0.03 [95% CI, −0.06 to −0.01]).

**Meaning:**

These results highlight the importance of hope and social support in understanding how financial strain relates to life satisfaction, providing a rationale for studying psychosocial approaches along with existing financial assistance strategies to reduce cancer-related financial toxicity.

## Teaser

This cross-sectional study of patients treated for cancer examines the association of financial toxicity burden with lower life satisfaction and hopefulness.

## Introduction

For over a decade, investigators have recognized that individuals with cancer experience financial toxicity, defined as the economic burden borne by patients with cancer and the family caregivers who accompany them.^1,2,3,4^ Up to half of those with cancer report financial toxicity, with multifaceted consequences: material burdens such as untenable out-of-pocket medical expenditures and interruption of income; impaired coping behaviors, including self-deprivation of food and medications or treatment nonadherence; and psychosocial distress.^5,6,7^

As metrics for financial toxicity emerged,^8,9,10^ initial intervention strategies primarily aimed to enhance health care delivery and material outcomes in order to improve patients’ ability to pay for treatment and receive care.^1^ Understandably, most intervention strategies emphasize mitigating the material burden of financial toxicity, concentrating on tools to increase patients’ economic resource access through financial education, medical cost discussions, and navigation to improve access to medication assistance, out-of-pocket payment plans, and optimized health insurance coverage.^11,12,13^ The effectiveness and limitations of available financial toxicity interventions were assessed in a systematic review and meta-analysis by Rashidi and colleagues.^14^ In their analysis, financial navigation strategies demonstrated an incremental but statistically significant impact on financial worry, but solely among the subset of patients with the most severe financial toxicity. For most patients, particularly those experiencing moderate financial toxicity, existing interventions lacked such effectiveness.

However, cancer-related financial toxicity impacts life beyond material burdens. The conceptual framework of financial toxicity posits equally vital psychological or psychosocial components.^15^ Studies support that financial toxicity is associated with worse psychological outcomes, including poorer health-related quality of life (HRQoL).^16,17,18^ However, such studies generally capture only health-focused aspects of individuals’ lives. Although cancer-related financial toxicity is a consequence of illness- and treatment-related cost stressors, financial toxicity may affect individuals’ well-being more broadly, including their overall satisfaction with life (SWL). Life satisfaction is a measure that integrates holistically across life domains. In studies not limited to medically ill populations, evidence suggests that greater financial burdens are associated with lowered overall life satisfaction, given that finances are necessary for most activities.^19,20,21^ Raising families, traveling, hobbies, education, and housing all require funds. Without resources, there may be little hope of attaining many of life’s cherished goals, whether struggling with the burdens of being medically ill or not. Therefore, for individuals with cancer, life satisfaction may serve as an ideally comprehensive and meaningful construct to measure the psychological toll of financial toxicity. Furthermore, identifying modifiable mediators of the psychological toll of financial toxicity is needed, given the limitations of current financial toxicity intervention strategies and the promise of harnessing internal and external psychosocial resources, especially when available material support diminishes.

Hope is a relevant potential mediator of psychological outcomes, given that it is related both to the pursuit of life’s goals and the establishment of life satisfaction.^22^ Although hope can sometimes be perceived as an amorphous phenomenon, it has been rigorously defined and measured through a model known as Hope Theory.^23^ Under this model, hope is measurable as a cognitive construct with 3 components: goals, pathways thinking, and agency thinking. Goals are strived-for ends. In the context of medical care, goals are often treatment-related; although it is noteworthy that, for most individuals, goals extend to many other domains (eg, relationships, work, hobbies). Pathways thinking encompasses plans or routes for achieving goals. Hopeful people tend to create multiple routes to navigate obstacles invariably encountered in pursuing goals. Finally, agency thinking denotes the thoughts that provide motivation for pursuing goals. Agentic thoughts (eg, “Even when things are difficult, I believe I can achieve my goals”) are often helpful when facing setbacks.

Validated instruments have been developed to quantify this construct of hopefulness,^24^ laying the groundwork to design, implement, and monitor interventions to enhance hope.^25^ Existing interventions aim to impart skills to increase the 3 components of hope, thereby impacting psychological resilience.^25,26^ Investigators have not yet used such interventions to target hopelessness related to financial toxicity, yet it is conceivable that hope-based interventions could harness an individual’s internal psychological capital to help mitigate the psychological effects of financial toxicity and manage the challenge of ongoing cancer-related financial worry, perhaps even before downstream damage to overall satisfaction with life becomes manifest.^27^

An additional candidate mediator in the association between financial toxicity and SWL is social support, a key modifiable external resource^28^ that could bolster internal resources to mitigate the psychological tolls of financial toxicity. In a literature review and empirical analysis of data from adults across the US, Pleeging et al^29^ found that degree of social support was robustly, positively associated with hope. Additionally, social support appeared to buffer the negative effects of cancer and cancer-related treatment on psychological well-being.^30,31^

Accordingly, in the present study, we sought to examine the association between financial toxicity and life satisfaction, hypothesizing that there is a direct association between these variables that is further mediated by social support and hope. This hypothesis flows from Hope Theory, which posits that obstacles to goal pursuit deplete hope and thereby lower general life satisfaction.^23^ Thus, cancer-related financial toxicity may impede hope by restricting perceived options and depleting motivation, thus undermining overall satisfaction with life. Social support may function as a resilience resource that reinforces hope by offering emotional encouragement, tangible aid, and informational guidance, which can help individuals reestablish hope for pursuing goals despite financial barriers. If such mediation were identified, this would support the study of interventions to augment these psychosocial assets as targets to improve financial toxicity–related psychological outcomes more effectively than current financial navigation strategies alone.

## Methods

### Participants and Procedure

The Economic Strain and Resilience in Cancer-II (ENRICh-II) study is a prospective, multicenter cohort study expansion from the previously reported ENRICh-I study of financial toxicity in patients and survivors with cancer.^4,9^ The present analysis represents baseline data from this study. In order to be included in the ENRICh-II study, participants were to be adults, have received ambulatory care for a pathologically confirmed cancer within a year prior, and have received care in 1 of the 6 oncology clinics (radiation, surgical, or medical) participating in the study. Among these facilities, 4 were strictly community-based centers, while the remainder subsumed both community and academic features (Table 1). Individuals were screened for eligibility between August 2020 and December 2022. Power analysis using Monte Carlo simulation^32^ estimated a necessary sample size of 251 to detect a small-to-medium association at 0.80 power. Of 1148 eligible patients invited by study coordinators, 605 consented, and 519 completed questionnaires, which were available in English or Spanish. Individuals provided written consent and the study was approved by MD Anderson Cancer Center institutional review board. This study followed the Strengthening the Reporting of Observational Studies in Epidemiology (STROBE) reporting guideline for cross-sectional studies.

**Table 1.** Study Sites

Site	Practice types	Size	Staffing
MD Anderson	Academic with community-based satellites	>3 Facilities	≥100 Staff
Baptist Health	Community-based	3 Facilities	50-99 Staff
DCH Health	Community-based	3 Facilities	≥100 Staff
Lyndon B Johnson Hospital	Community-based	2 Facilities	≥100 Staff
The Queens Medical Center	Community and academic	1 Facility	≥100 Staff
Scripps (satellite clinics)	Community-based	>3 Facilities	≥100 Staff

### Measures

#### Demographics

A demographics survey inquired about age, gender, race and ethnicity, income, and insurance status. These variables were gathered given their relevance to financial burden in previous research.^4^ Racial identity could be chosen from more than 800 descriptors and ethnic identity could be chosen from more than 40 descriptors. To enable statistical analysis, race and ethnicity were grouped into the following categories: American Indian or Alaskan Native, Asian or Pacific Islander, Black or African American, Hispanic or Latinx, White, and not reported. In addition, cancer diagnosis and whether the individual had received chemotherapy were gathered from patient records.

#### Financial Toxicity

Patients completed the 15-item Economic Strain and Resilience in Cancer (ENRICh) instrument,^9^ which yields scores for overall (or global) financial toxicity burden, along with subdomain scores for material burden, coping burden, and psychological distress. Global and subscale scores range from 0 to 10, with 0 representing the least financial toxicity burden and 10 representing the most severe. Prior studies identified a score of 5 or higher as indicative of severe financial toxicity.^33^ The material burden score reflects financial depletion, including out-of-pocket costs, spent savings, accumulated debt, and lost income related to cancer diagnosis, treatment, and survivorship. The coping burden score reflects the depletion of practical resources to cope with financial toxicity burdens, including savings or income, employment benefits, organization-based resources (eg, charities, professional organizations), and informal resources (eg, financial help from family).^34^ The ENRICh also includes a single item measuring financial toxicity–related psychological distress. Research demonstrates the reliability and validity of the ENRICh.^9,35,36,37^ In the present study, the ENRICh global scale had a Cronbach α = 0.93, material burden subscale α = 0.92, and coping burden subscale α = 0.83, indicating good to excellent internal consistency reliability.

#### Satisfaction with Life Scale (SWLS)

The SWLS^38^ is a measure of overall life satisfaction, with 5 items containing statements about life (eg, “In most ways my life is close to my ideal”) rated 1 (strongly disagree) to 7 (strongly agree). Research demonstrates the reliability and validity of the SWLS.^36^ In the present study, Cronbach α was 0.88, indicating good internal consistency reliability.

#### State Hope Scale (SHS)

The SHS^39^ was used to measure hope, with 6 items assessing agency and pathways thinking.^23^ It was designed to be sensitive to change over time, making it an appropriate measure of current level of hopefulness. Items include, “At the present time, I am energetically pursuing my goals,” and, “There are lots of ways around any problem that I am facing now,” which were rated 1 (definitely false) to 8 (definitely true). In the present study, Cronbach α was 0.92, indicating excellent internal consistency reliability.

#### Medical Outcomes Study (MOS) Social Support Survey

The MOS^40^ is a valid and reliable measure of perceived social support. It contains 19 items listing various aspects of the perceived availability of interpersonal emotional, informational, tangible, affectionate, and positive interaction support. Items include, “Someone you can count on to listen to you when you need to talk,” and, “Someone to help you if you were confined to bed.” Respondents indicate their perception of how often each type of support is available to them using a 1 (none of the time) to 5 (all of the time) scale. In the present study, Cronbach α = 0.92, indicating excellent internal consistency reliability.

### Statistical Analyses

Variables were examined for skewness and kurtosis. Pearson correlations, *t* tests, and ANOVA tests examined associations of financial toxicity with participant characteristics. Mediation analyses were performed using Hayes^41^ bootstrapping procedure (PROCESS 4.0) within the SPSS version 29.0 software package (IBM). This procedure, which allows for the simultaneous evaluation of the statistical effects of multiple factors, was used to test the abilities of social support and hope to account for variance in the association between financial toxicity and life satisfaction. Four multiple mediation models were tested, in which either the ENRICh global scale or 1 of its 3 subdomains served as predictor variable, SWLS served as criterion variable, and both the MOS and SHS served as serial mediating variables. These met established assumptions of regression-based path analysis,^42^ including linearity, independence of errors (ie, the residuals in each analysis were uncorrelated with one another), and lack of multicollinearity (ie, variance inflation factors below 2.0). Variables were screened for normality and covariates were included to account for potential confounding factors (age, gender, race and ethnicity, disease site, chemotherapy enrollment, employment, income, and insurance status). Indirect effects were estimated using a bootstrapping approach with 5000 resamples, providing 95% CIs. In each analysis, missingness was addressed through listwise deletion (complete-case analysis). Missing data were relatively minimal (10% to 11%), and patterns of missingness were consistent with random occurrence. The threshold for significance was *P* < .05 in 2-sided tests.

## Results

### Preliminary Analyses and Patient Characteristics

A total of 519 patients were included in the analysis (mean [SD] age, 52.0 [15.2] years; 349 female [67.2%]); they were relatively diverse in age, race and ethnicity, and cancer diagnosis (202 breast [38.9%]; 111 colon, rectum, or anal [21.4%], 65 biliary, liver, pancreas, or stomach [12.5%]) (Table 2). On univariate analyses, more severe financial toxicity was associated with younger age (global financial toxicity score: *r* = −0.39, *P* < .001; material burden subdomain: *r* = −0.31, *P* < .001; coping burden subdomain: *r* = −0.39, *P* < .001; and financial toxicity–related psychological distress: *r* = −0.33, *P* < .001). In addition, financial toxicity differed by race and ethnicity, with more severe financial toxicity in Black, Asian, and Hispanic individuals compared with non-Hispanic White individuals (global financial toxicity: *F*_5,498_ = 8.39, *P* < .001; material burden subdomain: *F*_5,502_ = 7.66, *P* < .001; coping burden subdomain: *F*_5,504_ = 7.72, *P* < .001; financial toxicity–related psychological distress: *F*_5,500_ = 6.77, *P* < .001). Lower scores on the material burden financial toxicity subdomain were found for those employed (either full or part time) compared with those without employment (*t*_506_ = −2.25, *P* = .01), but not on other aspects of financial toxicity (global financial toxicity: *t*_502_ = −1.19, *P* = .23; coping burden subdomain: *t*_508_ = 0.113, *P* = .91; financial toxicity–related psychological distress: *t*_502_ = −0.141, *P* = .89). Lower income was associated with greater financial toxicity burden (global: *r* = −0.27, *P* < .001; material burden subdomain: *r* = −0.19, *P* < .001; coping burden subdomain: *r* = −0.31, *P* < .001; financial toxicity–related psychological distress: *r* = −0.29, *P* < .001). Having insurance (private, employer-sponsored, Medicaid, or Medicare) was also generally associated with lower financial toxicity burden (global: *t*_502_ = 2.76, *P* = .006; material burden subdomain: *t*_506_ = 1.60, *P* = .11; coping burden subdomain: *t*_508_ = 3.10, *P* = .002; and financial toxicity–related psychological distress: *t*_504_ = 2.84, *P* = .002). Finally, more severe financial toxicity burden was associated with having received chemotherapy (global: *F*_1,501_ = 21.90, *P* < .001; material burden subdomain: *F*_1,505_ = 6.53, *P* = .01; coping burden subdomain: *F*_1,507_ = 41.04, *P* < .001; and financial toxicity–related psychological distress: *F*_1,503_ = 14.49, *P* < .001). There were no differences on any variable by gender. Thus, in subsequent analyses, we statistically controlled for age, race and ethnicity, disease site, chemotherapy enrollment, employment status, income, and insurance status.

**Table 2.** Patient Characteristics

Characteristics	Patients, No. (%) (N = 519)
Age, y	
18-24	28 (5.4)
25-34	49 (9.4)
35-44	76 (14.6)
45-54	123 (23.7)
55-64	123 (23.7)
65-74	87 (16.8)
75-84	29 (5.6)
≥85	1 (0.2)
Not reported	3 (0.6)
Gender^a^	
Female	349 (67.2)
Male	170 (32.8)
Other	0
Race and ethnicity	
American Indian or Alaska Native	2 (0.4)
Asian, Asian American, or Pacific Islander	34 (6.6)
Black or African American	49 (9.4)
Hispanic or Latinx	137 (26.4)
White	278 (53.6)
Not reported	19 (3.7)
Disease site	
Breast	202 (38.9)
Colon, rectum, anal	111 (21.4)
Biliary, liver, pancreatic, or stomach	65 (12.5)
Leukemia, lymphoma, or other hematologic	53 (10.2)
Uterine, cervix, or endometrial	14 (2.7)
Bladder, kidney, or prostate	11 (2.1)
Head and neck	9 (1.7)
Brain or other primary CNS	6 (1.2)
Lung	5 (1.0)
Other	43 (8.3)
Chemotherapy	
Yes	281 (54.1)
No	237 (45.7)
Unknown	1 (0.2)
Income, $	
0-9999	68 (13.1)
10 000-14 999	13 (2.5)
15 000-19 999	8 (1.5)
20 000-34 999	49 (9.4)
35 000-49 999	46 (8.9)
50 000-74 999	70 (13.5)
75 000-99 999	51 (9.8)
100 000-199 999	121 (23.3)
≥200 000	56 (10.5)
Not reported	37 (7.1)
Current employment status	
Yes (full- or part-time)	215 (41.4)
No	286 (55.1)
Not reported	18 (3.5)
Health insurance	
Private, employer-based, or marketplace	498 (96.0)
Uninsured	12 (2.3)
Not reported	9 (1.7)

Abbreviation: CNS, central nervous system.

^a^ Specified in the self-reported survey as gender with choices of male, female, and other.

Mean (SD) ENRICh financial toxicity scores were 4.24 (2.71) for the global scale, 4.83 (3.20) for material burden, 3.71 (2.72) for coping burden, and 5.41 (3.74) for financial toxicity–related psychological distress. Mean (SD) scores for satisfaction with life, hope, and social support were 24.87 (7.17), 34.95 (9.33), and 79.52 (21.63), respectively. All variables met assumptions of normality (kurtosis below 4, skewness below 2).^42^ There were significant correlations among all study variables when examining Pearson correlations, with greater financial toxicity burden consistently associated with lower levels of hope, life satisfaction, and social support (Table 3).

**Table 3.** Univariate Analyses: Pearson Correlations for Financial Toxicity, Social Support, Hope, and Satisfaction with Life Measures

Scale	ENRICh material		ENRICh coping		ENRICh psychological		Social support		Hope		Satisfaction with life	
	*r*	*P* value	*r*	*P* value	*r*	*P* value	*r*	*P* value	*r*	*P* value	*r*	*P* value
ENRICh global	0.92	<.001	0.93	<.001	0.81	<.001	−0.11	.01	−0.25	<.001	−0.34	<.001
ENRICh material	NA	NA	0.71	<.001	0.75	<.001	−0.15	<.001	−0.20	<.001	−0.28	<.001
ENRICh coping	NA	NA	NA	NA	0.69	<.001	−0.09	.05	−0.25	<.001	−0.32	<.001
ENRICh psychological	NA	NA	NA	NA	NA	NA	−0.15	<.001	−0.28	<.001	−0.34	<.001
Social support	NA	NA	NA	NA	NA	NA	NA	NA	0.31	<.001	0.34	<.001
Hope	NA	NA	NA	NA	NA	NA	NA	NA	NA	NA	0.57	<.001
Satisfaction with life	NA	NA	NA	NA	NA	NA	NA	NA	NA	NA	NA	NA

Abbreviations: ENRICh, Economic Strain and Resilience in Cancer survey; NA, not applicable.

### Hopefulness and Social Support as Mediating Factors of Financial Toxicity and Life Satisfaction

Following common conventions, βs of approximately 0.10, 0.30, and 0.50 are typically interpreted as small, medium, and large, respectively.^43^ In the first model, there were significant paths from global financial toxicity burden to both social support and hope (Figure). In turn, social support and hope each had significant paths to life satisfaction.

**Figure.**
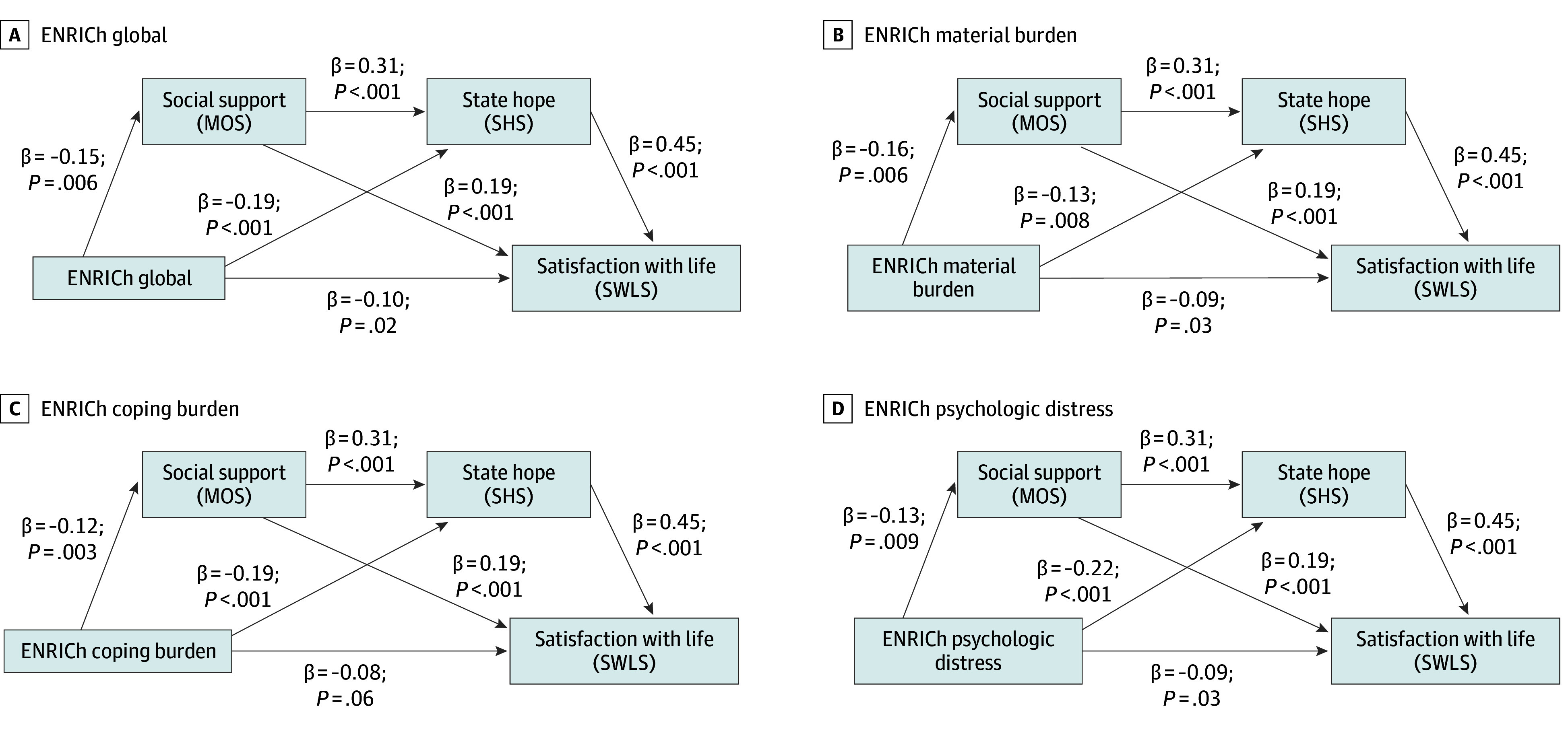
Multiple Mediation Models With Standardized Coefficients ENRICh indicates Economic Strain and Resilience in Cancer scale; MOS, Medical Outcomes Study Social Support Survey; SHS, State Hope Scale; SWLS, Satisfaction with Life Scale.

We then tested for statistical mediation within this model—that is, indirect associations between financial toxicity and life satisfaction through social support and hope. The simple indirect paths through social support (standardized effect = −0.03 [95% CI, −0.06 to −0.01]) and hopefulness (standardized effect = −0.08 [95% CI, −0.12 to −0.03]) were each significant, indicating that lower perceived social support and lower hopefulness separately mediated the association between greater financial toxicity burden and lower life satisfaction. Additionally, the multiple indirect path through social support and hopefulness together (standardized effect = −0.02 [95% CI, −0.04 to −0.01]) was significant, indicating that perceived social support and hopefulness are interrelated factors that combine to mediate the association between greater financial toxicity burden and lower life satisfaction. The fact that these indirect effects are negative indicates that higher financial toxicity was associated with lower levels of the mediating variables (hopefulness and social support), which in turn are associated with life satisfaction, consistent with expectations. Following common practices for interpreting indirect effects, magnitudes of 0.01, 0.09, and 0.25 are generally considered small, medium, and large, respectively.^44^ In this model, the path from financial toxicity burden to life satisfaction continued to be significant, indicating that there may be additional mediators of the association between financial toxicity and life satisfaction not measured in this study.

In the remaining 3 models, all financial toxicity subdomains demonstrated similar paths to life satisfaction through social support and hopefulness. For material burden, there were statistically significant paths to both social support and hopefulness. Each of these factors, in turn, manifested significant paths to life satisfaction. Tests for statistical mediation show that the simple indirect paths through social support (standardized effect = −0.04 [95% CI, −0.07 to −0.02]) and hope (standardized effect = −0.05 [95% CI, −0.09 to −0.01]), as well as the multiple indirect path (standardized effect = −0.03 [95% CI, −0.05 to −0.01]) were also significant. There was partial mediation, with the direct path from material financial toxicity burden to life satisfaction continuing to be significant. As with global financial toxicity, the negative value associated with the indirect effects indicates that greater material burden was associated with lower levels of the mediating variables (hopefulness and social support), which then were associated with life satisfaction, as expected.

For the subdomain of coping burden, there were significant paths to social support and hopefulness, each with significant paths to life satisfaction. Again, the simple indirect paths (social support: −0.02 [95% CI, −0.05 to −0.01]; hope: −0.08 [95% CI, −0.13 to −0.03]), and the multiple indirect path were also significant with partial mediation. As previously, the negative indirect effects indicate that higher levels of coping burden were associated with lower levels of the mediating variables (hopefulness and social support), which in turn were associated with life satisfaction.

Finally, for the subdomain of financial toxicity–related psychological distress, there were significant paths to social support and hopefulness, each with significant paths to life satisfaction. The simple mediational paths (social support: −0.04 [95% CI, −0.07 to −0.02]; hope: −0.09 [95% CI, −0.14 to −0.05]) and the multiple mediational path (−0.02 [95% CI, −0.04 to −0.01]) were also significant with partial mediation. As with the previous analyses, the negative indirect effects indicate that greater financial toxicity–related psychological distress was associated with lower levels of the mediating variables (hopefulness and social support), which then were associated with life satisfaction.

## Discussion

Results from this multicenter analysis demonstrate direct and indirect associations of financial toxicity with hopefulness, perceived social support, and life satisfaction in patients with cancer. Specifically, in a relatively large and diverse sample, levels of hopefulness and perceived social support played a mediating role in the association between financial toxicity and life satisfaction, with greater financial toxicity burdens (across subdomains) associated with lower life satisfaction.

These findings add to the growing evidence delineating psychological and psychosocial factors impacting patient outcomes, underscoring the importance of addressing financial toxicity alongside variables such as hope^25,26^ and social support^28^ as aspects of comprehensive cancer care. Future investigation may explore the effects of interventions to augment hope, social support, or other potential mediators (eg, resilience, gratitude) on psychological well-being, but also possibly on the adverse economic and health outcomes associated with financial toxicity.

Our analyses show that hope and social support statistically mediate (ie, explain variance in) the association of higher financial toxicity with lower life satisfaction. Although the results cannot definitively establish causality given that the present study did not involve manipulating financial strain (or resources), hope, or social support, they nonetheless suggest that interventions enhancing hope and social support may help alleviate the negative impact of financial toxicity on overall life satisfaction. This provides a future basis to empirically test such interventions, with the goal of improving the psychological well-being of a substantial portion of individuals facing financial burdens and their downstream effects. Both multisession^45,46^ and single-session^25,26^ hope interventions have demonstrated effectiveness for developing skills for nurturing goals, pathways, and agency in the context of social support. Such psychosocial interventions could be tested in tandem with screening for financial toxicity and referral to financial counselling, which alone have not demonstrated sufficient effectiveness for alleviating the worry and burden of financial toxicity in recent systematic analyses.^14^

These findings also contribute conceptually to understanding the mechanisms linking economic hardship and psychological well-being, extending Hope Theory^23,24^ into a cancer care context. According to Hope Theory, hope involves the motivation to pursue valued life goals and the perceived capacity to identify routes toward them. In the present study, greater financial toxicity was associated with lower levels of both hopefulness and social support, which in turn were associated with life satisfaction. This pattern suggests that economic strain may undermine patients’ perceived ability to set and pursue meaningful goals, while also constraining their sense of connectedness and available support. Conversely, increases in hope and social support may reinforce and expanding perceived options, which are associated with greater overall life satisfaction.

### Limitations

This study has several limitations. Our cross-sectional baseline analysis from the ENRICh-II cohort provides a snapshot of the association of financial toxicity with hope, social support, and life satisfaction in a relatively diverse sample across a variety of cancer diagnoses and care settings, but stops short of proving causality. The present research provides evidence of statistical mediation—that is, both social support and hope account for significant variance in the association between financial toxicity and satisfaction with life. Nonetheless, we caution against inferring causality in the absence of prospective data. Additionally, although this study’s use of patient-reported measures was necessary for assessing subjective experiences like hope and life satisfaction, these could be affected by recall or social desirability biases. Relatedly, the association among these constructs could be somewhat inflated by common method variance, given that all variables were assessed through questionnaires. Thus, our results may be further validated using both longitudinal designs and mixed methods approaches (perhaps including direct behavioral observation or physiological measures of emotion) to provide additional insights into the personal experiences of hope, social support, and life satisfaction as related to financial toxicity.

Finally, as with all studies, unmeasured variables may contribute to the observed association of financial toxicity with hope, social support, and life satisfaction. Future studies could include measures of additional psychosocial variables (eg, coping styles, resilience, or perceived control) that may be linked with both financial strain and hopefulness. External variables such as access to financial counselling or clinician communication quality may also play roles. Nonetheless, our inclusion of multiple relevant covariates and a theoretically guided model reduce the likelihood that uncontrolled confounding fully accounts for the observed results.

## Conclusions

This study highlights psychological supports combined with financial navigation as potential critical components of comprehensive cancer care, in a population in which nearly half of individuals report financial toxicity. During the past 2 decades, multiple successful examples of interventions designed to augment hope and social support have been developed to address an array of outcomes^25,44^ and, with continued study, it is entirely plausible that financial toxicity can be added to that list.

## Notes

Supplement. Data Sharing Statement
