# Supplementary material for: Financial Toxicity, Hope, and Satisfaction With Life in Patients Receiving Ambulatory Cancer Care
Source: JAMA Netw Open. 2026 Feb 5;9(2):e2557328. doi: 10.1001/jamanetworkopen.2025.57328 (PMC12878433; doi:10.1001/jamanetworkopen.2025.57328)
Supplement: Supplement. — Data Sharing Statement [file jamanetwopen-e2557328-s001.pdf]

Data Sharing Statement

Smith. Financial Toxicity, Hope, and Satisfaction With Life in Patients Receiving Ambulatory Cancer Care. *JAMA Netw Open*. Published February 05, 2026.
doi:10.1001/jamanetworkopen.2025.57328

Data
Data available: No
